# Regulation of inner nuclear membrane associated protein degradation

**DOI:** 10.1080/19491034.2019.1644593

**Published:** 2019-07-25

**Authors:** Bailey Koch, Hong-Guo Yu

**Affiliations:** Department of Biological Science, The Florida State University, Tallahassee, FL, USA

**Keywords:** INMAD, ERAD, APC/C, SUN-domain protein, E3 ubiquitin ligase, ubiquitin-proteasome system, ER-phagy, nucleophagy, nuclear envelope, and laminopathy

## Abstract

The nucleus is enclosed by a double-membrane structure, the nuclear envelope, which separates the nucleoplasm from the cytoplasm. The outer nuclear membrane is continuous with the endoplasmic reticulum (ER), whereas the inner nuclear membrane (INM) is a specialized compartment with a unique proteome. In order to ensure compartmental homeostasis, INM-associated degradation (INMAD) is required for both protein quality control and regulated proteolysis of INM proteins. INMAD shares similarities with ER-associated degradation (ERAD). The mechanism of ERAD is well characterized, whereas the INMAD pathway requires further definition. Here we review the three different branches of INMAD, mediated by their respective E3 ubiquitin ligases: Doa10, Asi1-3, and APC/C. We clarify the distinction between ERAD and INMAD, their substrate recognition signals, and the subsequent processing by their respective degradation machineries. We also discuss the significance of cell-cycle and developmental regulation of protein clearance at the INM, and its relationship to human disease.

## Introduction

**Figure 1.**
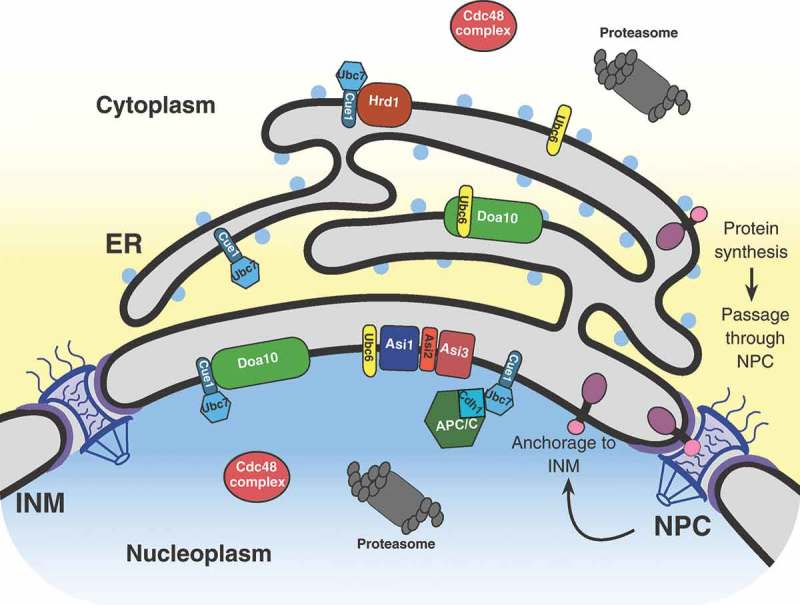
Schematic representation of a eukaryotic cell, focusing on the nucleus, nuclear envelope, and ER. This demonstrates the current view of integral INM protein synthesis within the ER, subsequent passage through the nuclear pore complex (NPC), and final anchorage to the INM. Additional proteins and complexes pertinent to this review are also shown in their native locations. ER, endoplasmic reticulum; INM, inner nuclear membrane.

The eukaryotic nucleus is enclosed by a double-membrane structure, the nuclear envelope, that separates the nucleoplasm from the cytoplasm. The outer nuclear membrane (ONM) is continuous with the endoplasmic reticulum (ER), whereas the inner nuclear membrane (INM) is biochemically distinct, partitioned from the ONM by nuclear pore complexes (NPC) [1,2]. The INM is a specialized compartment within the cell with a unique proteome and the ability to synthesize lipid droplets [3]. Nascent INM proteins are synthesized in the cytoplasm or the ER before passage through the NPC by either an energy-dependent or diffusion-retention mechanism, and then anchored at the INM (Figure 1, [4,1]). There are three distinct classes of proteins targeted to the INM: peripheral INM proteins anchored through protein-protein interactions, peripheral INM proteins associated with the outer leaflet of the INM through amphipathic helices/post-translational modifications, and integral INM proteins [5]. INM proteins are crucial for regulating a wide range of nuclear activities that include chromosome movement, gene expression, and signal transduction [6]. Abnormal accumulation of INM proteins, such as the integral membrane protein SUN1, has been linked to the pathogenesis of progeric and dystrophic laminopathies in mammals [7,8]. The INM-associated degradation (INMAD) is required for both protein quality control and regulated proteolysis of INM proteins. INMAD shares similar biochemical properties with ER-associated degradation (ERAD). In addition to INMAD, selective autophagic processing has also been reported for protein turnover at the INM through macro and micronucleophagy, including piecemeal nucleophagic processes [9,10]. Here we focus on the three distinctive branches of the INMAD pathway for INM protein turnover.

## The ubiquitin-proteasome system for protein turnover

The most common mechanism for protein quality control and degradation within eukaryotic cells is the ubiquitin-proteasome system [11,12]. This mechanism functions through the conjugation of ubiquitin polymers to a target protein, a process termed ubiquitylation, which acts as a recognition signal for destruction by the 26S proteasome [12]. Generally, ubiquitylation follows three sequential steps. First, the C-terminal carboxyl group of a ubiquitin molecule is activated through an ATP-dependent reaction with a ubiquitin-activating enzyme (E1). The ubiquitin is transferred to a ubiquitin-conjugating enzyme (E2) which then binds to a ubiquitin ligase (E3). The ubiquitin is then transferred to a lysine sidechain of the substrate either from the ubiquitin present on the E3 directly or in an E3 mediated transfer from the E2, marking it for degradation [11]. Atypical ubiquitylation on serine/threonine residues has also been found for some proteasomal substrates [13–15]. The E3 enzymes are the largest class within the ubiquitylation pathway, containing approximately 100 E3s in budding yeast and 600 in humans [16,17]. Consequently, E3 enzymes are the most critical component responsible for substrate recognition and specificity within the ubiquitin-proteasome system.

## ERAD degrades ER-associated proteins

**Figure 2.**
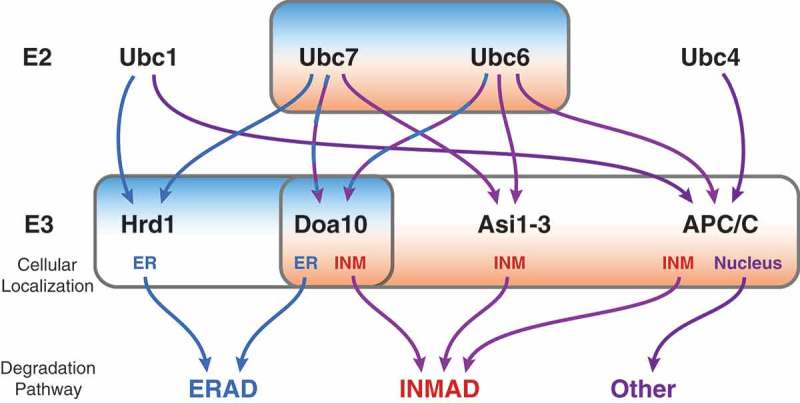
Flow chart comparing ERAD and INMAD. Ubc6 and Ubc7 are the most commonly utilized E2s for both ERAD and INMAD, functioning with either Hrd1 or Doa10 for ERAD, or Asi1-3, APC/C, or Doa10 for INMAD. Ubc1 can also function as an E2 for ERAD with Hrd1, and with APC/C in an unrelated context. Ubc4 also functions with APC/C outside INMAD.

Due to the high rate of protein synthesis at the ER, an assortment of regulatory factors is present to ensure proper folding and modification of nascent proteins [18]. Proteins that fail to fold properly, are not correctly modified, or are in excess of their proper stoichiometry are subject to protein quality control through the ERAD pathway [19,20]. In the budding yeast *Saccharomyces cerevisiae*, there are two ER membrane-resident E3 ligase complexes, centered on the E3 ligases Doa10 and Hrd1, representing two independent branches of ERAD. These two canonical ERAD branches are responsible for the degradation of substrates with misfolded domains or degrons within the ER lumen (ERAD-L), membrane spanning region (ERAD-M), cytoplasm (ERAD-C), and the more recently discovered translocon-associated region (ERAD-T) [21–24]. Two partially redundant E2 enzymes, Ubc6 and Ubc7, the latter bound to Cue1, serve as the canonical ubiquitin conjugating enzymes within ERAD [19,25,26] functioning with Doa10 [19,24]. The Hrd1 complex functions mainly with Ubc7, and additionally with Ubc1 (Figure 2 [27,28]).

Interestingly, Ubc6 and Ubc7’s enzymatic activity is not unique to E3 ligases located at the ER, as the Doa10 complex has been found to function at the INM in conjuction with Ubc6 and Ubc7 (Figures 1 and 2; [29,30]). Additionally, recent studies have identified novel functions of the INM transmembrane Asi1-3 complex, and the soluble nuclear anaphase-promoting complex/cyclosome (APC/C) as E3 ligases working in conjunction with Ubc6 and Ubc7 to regulate protein turnover of substrates specifically at the INM (Figure 2; [31-34]). These spatially compartmentalized pathways have been termed INMAD for Inner Nuclear Membrane Associated Degradation [35]. Although initially designated as separate ERAD branches, for the purpose of this review both the Asi1-3 complex and Doa10 functioning at the INM will be referred to as INMAD (Figure 2).

## INMAD degrades INM-associated proteins

The term INMAD was first coined to describe the mechanism of degradation of Asi1, a subunit of the Asi1-3 complex [35]. However, previous studies in budding yeast documented proteasomal targeted degradation of nuclear- and membrane-bound substrates by INM localized E3 ubiquitin ligases, Doa10 and the Asi1-3 complex [29–32], and INMAD has since been expanded to include the APC/C [33]. Current knowledge dictates INMAD has many mechanistic similarities to ERAD in the sense that both to some degree utilize the E2 conjugating enzymes Ubc6 and Ubc7 and also the AAA-ATPase, Cdc48, prior to degradation by either nuclear or cytoplasmic proteasomes, respectively [29–33]. Whereas the different branches of ERAD have been extensively studied and have well-defined mammalian homologs, the branches and regulatory factors of INMAD remain to be elucidated. Two branches of the INMAD pathway, Doa10 and APC/C, appear to be onserved in mammals [29,33]. However, the plethora of proteins present at both the budding yeast and mammalian INM is highly diverse, allowing one to speculate that there may be more INMAD substrates for the currently defined pathways and potentially more ubiquitin ligases as well.

## Doa10 complex and INMAD

The Doa10 complex is an E3 ubiquitin ligase present and active at both the ER and INM and is therefore part of both ERAD and INMAD (Figure 2). Doa10 was identified initially as a novel RING finger E3 that ubiquitinates the extremely short-lived transcription factor, MATalpha2, in the nucleus [12,24]. Doa10 is believed to passively diffuse from the ER to the INM through the NPC, despite not containing an obvious nuclear localization signal [29]. Doa10’s localization to the INM is pertinant for nuclear (INMAD) substrate degradation [29]. Doa10-mediated ERAD and INMAD functions with the E2 conjugating enzymes Ubc6 and Ubc7 for substrate ubiquitylation prior to cytoplasmic or nucleoplasmic proteasomal degradation, respectively [24,29]. Doa10 substrates embedded in the membrane require the AAA-ATPase Cdc48 complex for degradation, whereas soluble substrates do not [36]. This is likely due to the role of the Cdc48 complex in membrane extraction of substrates.

Doa10-mediated ERAD substrates contain destruction motifs, also called degrons that are recongized by the E3 ligase, exposed to the cytoplasm, therefore called ERAD-C. Two Doa10-dependent degrons, Deg1 and CL1, have been extensively characterized. Interestingly, both consist of amphipathic α-helices [36]. Deg1 represents the first 67 residues of MATalpha2, and recognition by Doa10 is proposed to be regulated by N-terminal acetylation and N-terminal methionine excision [37–39]. Doa10-mediated nuclear substrates include the transcription factor MATalpha2, kinetochore component Ndc10, the integral INM protein Asi2, as well as other soluble and membrane-bound nuclear proteins [29,30,36]. This branch of INMAD has been well studied, and many speculations regarding substrate selection due to nucleoplasmic folding lesions and aggregation propensity can be inferred from Doa10-mediated ERAD [21,40,41]. However, the specific rules that define substrate selection for this branch of INMAD remains to be further elucidated.

## Asi1-3 complex and INMAD

Originally, the *ASI* genes were identified as amino acid sensor-independent genes that negatively regulate the amino acid sensing pathway by degrading transcription factors Stp1 and Stp2 [42]. The Asi1-3 complex recognizes unprocessed full-length Stp1 that inappropriately reaches the nucleus by virtue of the Stp1 N-terminal conserved region, termed R1 [43]. The Asi1-3 complex subsequently targets this transcription factor for nuclear proteasomal degradation by polyubiquitinating this N-terminal regulatory domain of Stp1 [43–45].

The Asi1-3 complex is largely composed of transmembrane-domain proteins Asi1, Asi2, and Asi3, with the characteristic RING finger E3 ligase domain presents on the C-termini of both Asi1 and Asi3 [43,45]. Asi2 does not have a domain with ligase activity, but is in complex with Asi1 and Asi3 at the INM. Asi2 is not required for the formation of the Asi1-3 complex, but is instead thought to function as an adapter needed for substrate recognition [31,46]. Within INMAD, the Asi1-3 complex has been shown to function with the E2 conjugating enzymes Ubc6 and Ubc7 for substrate ubiquitylation [31,32]. After ubiquitylation, the substrate is likely extracted from the membrane by the activity of the complex containing Cdc48, prior to degradation by nuclear proteasomes [31]. However, the mechanism and the specific degron motifs mediating membrane extraction and destruction for Asi-mediated INM protein turnover have yet to be elucidated. The lack of a clear degron consensus sequence may indicate that Asi1-3 is primarily invovled in degrading misfolded or mislocalized proteins at the INM as a means of protein quality control [31,32].

INMAD substrates of the Asi1-3 complex were first found through genome-wide analyses of the yeast proteome utilizing either a tandem fluorescent timer or quantitative mass spectrometry-based approach [31,32]. These analyses identified the sterol biosynthesis proteins Erg11 and Nsg1, the vacuolar proteins Vcx1, Vtc1, and Vtc4, and other membrane proteins as INMAD substrates [31,32]. Each of these proteins exhibited a pronounced increase in half-life and INM accumulation in an *ASI1* mutant background [31,32]. More recently, additional native Asi-mediated INMAD substrates have been found to include the LEM-domain protein Heh2 and the nucleoporins Pom33 and Pom34 [34]. In this case mutation of *ASI1* did not necessarily change protein stability but instead affected protein nuclear distribution, providing evidence for another role of INMAD in regulating protein localization [34]. As mentioned previously, there is no known degron motif for the Asi1-3 complex; however, the identification of additional Asi1-3 substrates at the INM will assist revealing the consensus motif, if there is one. Notably, exposure of hydrophobic surfaces that are normally buried within folded proteins is critical for turnover of many misfolded cytosolic protein quality control and ERAD substrates [47]; such a shared recognition mechanism could conceivably mediate turnover of misfolded INM proteins as well.

## APC/C and INMAD

APC/C is a large E3 ligase complex originally determined to be responsible for controlling cell-cycle progression through the ubiquitination of cyclins and subsequent proteasomal degradation in both budding yeast and higher eukaryotes [48–50]. The cognizant E2 enzymes functioning with APC/C are Ubc1 and Ubc4 in budding yeast [17,51]. Two activators, Cdc20 and Cdh1, regulate APC/C activity throughout the cell cycle [52]. These activators recognize specific destruction motifs, and therefore most canonical APC/C substrates have been well characterized as containing D- and/or KEN-box consensus sequences [53,54].

**Figure 3.**
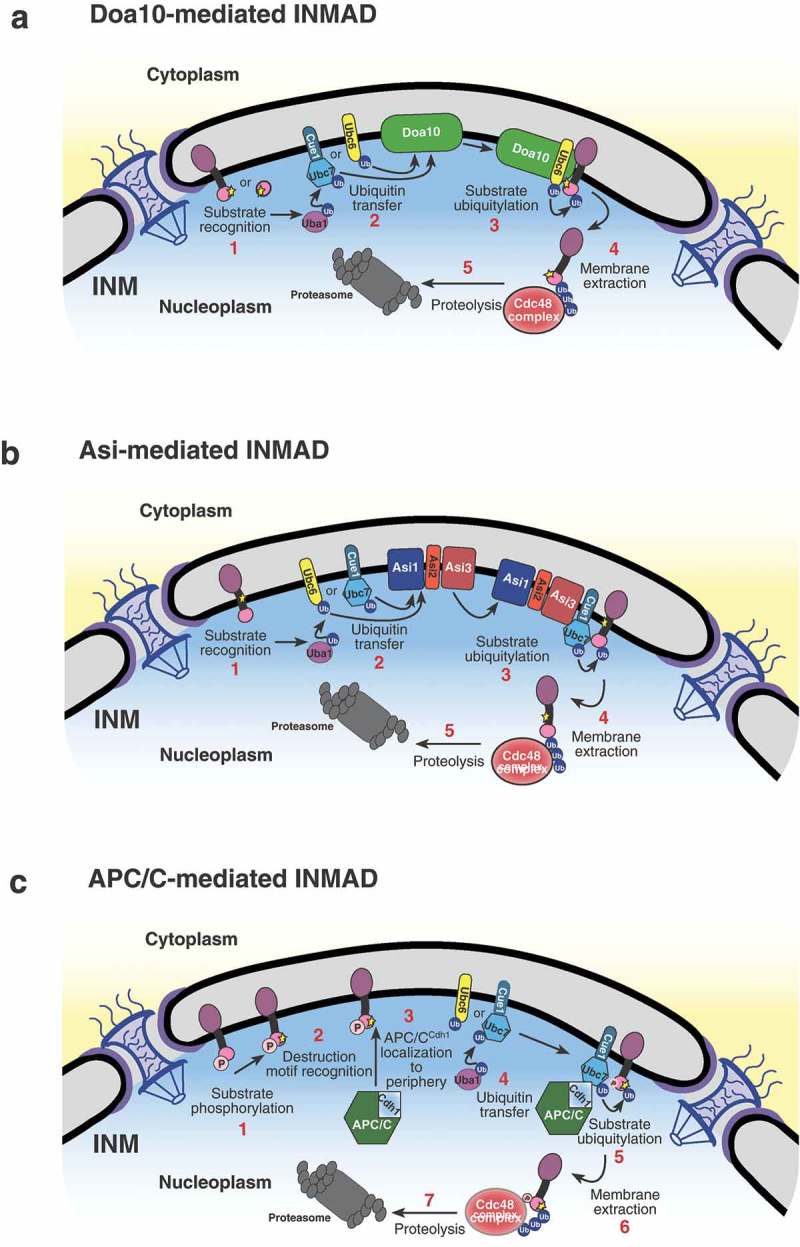
Models for INMAD-mediated protein degradation. (A) Substrates with a nucleoplasmic degradation signal are recognized, and the E1 enzyme Uba1 initiates the ubiquitin cascade. Uba1 transfers ubiquitin to either Ubc6 or Cue1-bound Ubc7, which associates with and relays the ubiquitin to the E3 ligase Doa10. Doa10 in complex with either Ubc6 or Ubc7/Cue1 then catalyzes the transfer of ubiquitin to the substrate, targeting it for membrane extraction by Cdc48 and subsequent proteasomal degradation. (B) Substrate recognition for Asi-mediated INMAD is currently unclear. However after the protein is recognized for degradation, Uba1 initiates the ubiquitin cascade and association with Ubc6 or Ubc7 as described in A, but utilizing the Asi1-3 complex. (C) Substrates with a KEN and/or D-box destruction motif exposed to the nucleoplasm are likely phosphorylated and recognized by the Cdh1-activated APC/C, causing the APC/C to localize to the nuclear periphery. The E1 enzyme Uba1 then initiates the ubiquitin cascade, transferring ubiquitin preferentially to Ubc7/Cue1. Ubc7/Cue1 in conjunction with APC/C^Cdh1^ then catalyze the transfer of ubiquitin to the substrate, targeting it for membrane extraction by Cdc48 and subsequent proteasomal degradation.

**Figure 4.**
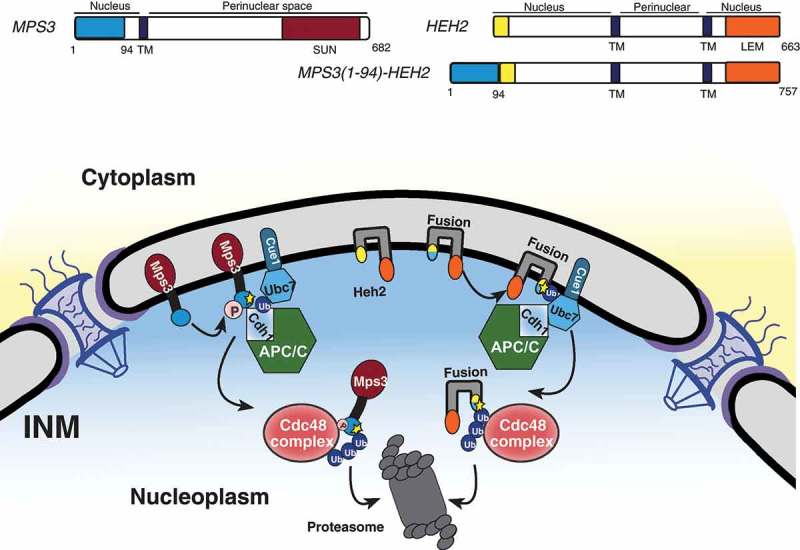
The N terminus of Mps3 is sufficient for APC/C-mediated INMAD. (A) A schematic representation of INM proteins Mps3 and Heh2, and the fusion protein Mps3(1–94)-Heh2 protein structure and membrane topology. TM, transmembrane domain. (B) Mps3 recognition for degradation by APC/C proceeds as described in Figure 3C. Mps3(1–94)-Heh2 (Fusion) behaves similarly, showing a dependence on the N terminus of Mps3 for recognition by APC/C and subsequent degradation. INM protein Heh2 is endogenously stable.

We have shown that the APC/C represents another branch of the INMAD pathway operating to degrade the resident INM protein Mps3 (Figure 3(c)). Mps3 is a SUN-domain protein within the functional linker of the nucleoskeleton to cytoskeleton (LINC) complex and is essential for proper spindle pole body duplication and insertion in budding yeast [33,55]. The N-terminus of Mps3 was also recently found to be essential for spindle pole body separation and adequate cell-cycle progression [33,56]. This same N-terminal region contains a crucial phosphorylation site, serine 70, and two putative APC/C destruction motifs found to be required for protein turnover regulated by Cdh1 [33]. Additionally, a fusion protein consisting of the otherwise stable INM protein Heh2 with Mps3’s two putative destruction motifs appended to its N-terminus is rapidly degraded, implying that these motifs are not only necessary but also sufficient to induce APC/C-mediated INMAD (Figure 4; 33). Other proteins associated with the INM, including Dia2 and Sae2 [31], may contain putative D or KEN box motifs (our unpublished data); whether they are subject to APC/C regulation is currently unknown.

APC/C-mediated INMAD likely begins with substrate phosphorylation and subsequent destruction motif recognition through the Cdh1-activated APC/C. However, the exact initiating steps are unclear. APC/C appears to function with the canonical INMAD E2 conjugating enzyme Ubc7, and to a lesser degree Ubc6, to transfer ubiquitin to Mps3. We speculated that ubiquitinated Mps3 is then extracted from the membrane by the AAA-ATPase Cdc48 complex and transferred to nuclear proteasomes for proteolysis [33]. The inhibition of any of these steps results in a pronounced increase in Mps3’s half-life, providing a definitive degradation pathway for a resident INM protein [33].

Unlike the integral Doa10 and Asi1-3 complexes, APC/C is a soluable protein complex best characterized in its role in degrading soluble nuclear proteins in budding yeast [29,43,57,58]. It must be recruited to the nuclear periphery because it does not possess any membrane components. The recruitment of the APC/C to the INM periphery has yet to be defined, but we speculate that the APC/C-dependent ubiquitylation of INM proteins is enhanced through substrate phosphorylation [33,59] as the crosstalk between ubiquitylation and protein phosphorylation is common for known APC/C substrates [58,60,61]. Alternatively, Cdh1, the APC/C activator, may be recruited to the INM and therefore the APC/C may follow. The discovery of more APC/C-mediated INMAD substrates will yield a clearer understanding of this process.

## Retrotranslocation of ERAD substrates by Cdc48: a clue for INMAD

After or simultaneously to ubiquitylation, ERAD substrates are retrotranslocated from the ER into the cytoplasm for proteasomal degradation [62–64]. In most cases, this process is energy-dependent upon the AAA- ATPase Cdc48, which forms a hexameric ring and binds to cofactors that include Ufd1 and Npl4 [63,65,66]. There are multiple methods for transporting soluble, integral, and transmembrane helix (TMH)-containing proteins to the cytoplasm. Early studies have shown that Sec61 and Sec63 play an important role in substrate translocation [67–69], more recently, evidence was found of ERAD machinery, specifically Der1 and Hrd1, forming a proteinaceous channel allowing ER luminal substrates to pass into the cytoplasm [70].

Recent biochemical and structural analyses of purified Cdc48 components have revealed mechanistic insights into substrate extraction [71]. First, Cdc48 in complex with Ufd1 and Npl4 appears to bind the ubiquitylated substrates at the face of the ring complex containing the Cdc48 N-terminal domains [65,72–74]. Then, the Cdc48’s ATPase activity drives substrate unfolding, allowing passage of the unfolded polypeptide through the central pore of the Cdc48 complex, followed by substrate proteolysis [75,76]. Finally, the deubiquitylating enzyme Otu1 is required for substrate release after it unfolds and begins transfer through the Cdc48 pore [71,77].

Not all integral ER proteins, however, are subject to Cdc48-mediated retrotranslation; some remain embedded and form a complex directly with the proteasome, in a process mediated by the Cdc48 complex [78]. Due to the similarities between E2 conjugating enzymes, and the requirement for Cdc48 in the described INMAD pathways, it is likely that a similartranslocation process occurs at the INM to transfer substrates into the nucleoplasm [31,33,35]. Further analysis of the molecular mechanisms underlying INMADsubstrate translocation is required for a more mechanistic understanding of this process.

## Autophagy-dependent protein degradation at the ER and INM

Other than ERAD and INMAD, there is a proteasome-independent method of protein clearance at the outer and inner nuclear membranes that occurs through autophagy [9]. This autophagic process can occur through piecemeal, or micro, nucleophagy or macronucleophagy [9]. Autophagy is the engulfment of a small volume of intracellular components for degradation, used to regulate a variety of cellular functions. Perturbation of autophagy is closely linked to several human diseases, including but not limited to osteoarthritis in the liver and neurodegenerative diseases such as Huntington and Alzheimers [79–81]. In the autophagic clearance mechanism, receptor proteins recognize selected cargo and direct their sequesteration by a double-membrane structure, the autophagosome, which then fuses with the lysosome (or vacuole in budding yeast) where its contents are degraded by resident hydrolases [82,83]. Formation of the autophagosome is largely dependent upon the ubiquitin-like proteins Atg12 and Atg8 [84]. Atg12 undergoes a series of reactions to yield an Atg12-Atg5 conjugate. This conjugates then binds Atg16 to form an E3-like complex that stimulates the conjugation of Atg8 to the lipid phosphatidylethanolamine (PE, Atg8-PE) [85,86]. In selective autophagy, autophagy receptors bind to the Atg8-PE conjugates and are crucial for forming autophagosomal membranes to allow membrane expansion along the surface of degradation targets [82,87]. Selective autophagy has been characterized in the quality and quantity control of various cellular components, such as the mitochondria and peroxisomes, and more recently the ER and nucleus [10,88–90]. Atg39 and Atg40 are the budding yeast autophagy receptors found to mediate ‘nucleophagy’ and ‘ER-phagy;’ they localize to the nuclear membrane, including the INM and ONM, and cortical ER, respectively, in response to nitrogen starvation [10]. Both ER-phagy and nucleophagy have been described in response to tumorigenic stresses in mammalian cells [91,92]. These observations indicate that ER-phagy and nucleophagy, of both the INM and ONM, are important degradation systems for targeting ER and nuclear components in response to various cellular stresses. However, the molecular mechanisms overseeing these processes are largely unknown.

## Cell-cycle and developmental regulation of protein turnover at the INM

The timing of protein turnover is crucial in various cellular compartments for a multitude of reasons, and turnover of resident proteins at the INM is no different. APC/C-mediated degradation of the resident INM protein, Mps3, appears to function in a cell-cycle dependent manner [33]. Due to the cyclical nature of APC/C activation by Cdh1 or Cdc20 throughout different mitotic stages, it makes sense that APC/C substrates’ turnover is coupled to cell-cycle progression [48,52,93–95]. The reported cell-cycle dependence is consistent with the function of APC/C-mediated INMAD substrate Mps3’s function in spindle pole body duplication and separation in budding yeast [33,55,56,96]. These observations support the idea that APC/C-mediated INMAD regulates the budding yeast SUN-domain protein, and potentially other essential INM proteins’ turnover during the cell cycle.

Highly conserved SUN proteins compose the nuclear side of the LINC complex, which spans the nuclear envelope through the SUN/KASH-protein interaction. One crucial function of the LINC complex is ensuring proper nuclear movement. Nuclear movement is critical for neurogenesis and myogenesis [97–99]. In mice, SUN and KASH proteins play essential roles in neurogenesis and neuronal migration by mediating centrosome-nucleus coupling during both interkinetic nuclear migration and radial neuronal migration in the developing cerebral cortex [99]. These proteins are also important for nuclear anchorage and nuclear movement [99], similar to their function observed in the distribution of myonuclei along the cell membrane in muscle cells [100]. In addition to the mouse model, SUN and KASH proteins regulate the distribution of nuclei throughout muscle cells in drosophila embryos and larvae, and in cultured mammalian cells [97,101]. That these nuclear-envelope proteins are required for the correct dissociation of nuclei during the development of multiple tissue types [98], invites speculation into the conserved significance of protein turnover in both the inner and outer nuclear membranes.

## The INM and human disease

Since the discovery of the nuclear envelope, knowledge about its structure and function has increased significantly through the development of a multitude of assays capable of visualizing this organelle. Research on the INM is relevant to human health due to the effects of mutations in genes encoding lamins, some INM proteins, and other associated proteins, causing a wide range of diseases referred to as laminopathies [62,102]. In mammalian cells harboring mutations in nuclear lamina genes, specifically LMNA and LMNB1, autophagosomes have been found to contain these nuclear components, implying a potential role for autophagy in the clearance of unwanted INM-associated proteins in laminopathic or oncogenic cells [91,92]. Additionally, the budding yeast SUN-domain protein Mps3 has been reported to accumulate at the INM, resulting in nuclear envelope expansion [96] and impaired cell cycle progression [33,56]. Mps3 is homologous to SUN1, whose accumulation at the INM has been implicated in progeric and dystrophic laminopathies [7]. In addition, the lamin B receptor (LBR), another INM protein, is subject to the regulation by the ubiquitin-proteasome system inside the nucleus, whether LBR is a substrate of INMAD remains to be determined [103]. Elucidating the conserved nature of INMAD processes in budding yeast is crucial to understanding many laminopathies in humans.

## Conclusions and future directions

We have discussed the mechanisms crucial for protein clearance from the nuclear envelope through pathways mediated by INMAD, ERAD and autophagy (ER-phagy and nucleophagy), processes which likely interplay. ER-phagy and nucleophagy are known to be key processes in the initiation of meiosis for the clearance of unwanted proteins in budding and fission yeast [104,105]. Nucleophagy has also been reported in animal cells with mutagenized lamins and cells that have been exposed to specific oncogenic stresses [91,92]. However, the molecular mechanisms governing the autophagic stress response for these processes have yet to be well defined.

In comparison to INMAD, ERAD is a well-defined degradation pathway for protein quality control within the ER. The majority of the ERAD machinery for substrate recognition, ubiquitylation, and retrotranslocation has been well described in both budding yeast and mammals. Currently INMAD is known to have at least three branches in budding yeast: the Doa10 complex, the Asi1-3 complex, and APC/C, which function with Ubc6 and Ubc7 to target INM substrates for proteasomal degradation [31–33]. Two of the E3 ligases within INMAD, Doa10 and APC/C, have known mammalian homologs, whereas the Asi1-3 complex does not [29,31–33]. The highly conserved nature of both the APC/C and the budding yeast SUN-protein Mps3 suggest a mechanism of regulation previously undescribed for mammalian SUN1 [33]. The recent expansion in knowledge of E3 ligase activity at the INM has caused more questions to be raised about the mechanisms of INM proteostasis through INMAD. Future research will provide insight into how Ubc6 and Ubc7 are partitioned between Doa10, Asi1-3, and APC/C, and the conserved nature of each of the discussed INMAD branches, their mechanism of substrate recognition, and the exclusivity of these independent pathways.
